# Wound infection and subsequent port-site hernia following laparoscopic appendectomy: A case report and surveillance data analysis

**DOI:** 10.1016/j.ijscr.2022.107235

**Published:** 2022-05-25

**Authors:** Katie Jeddeloh, Jena Velji-Ibrahim, Emily Stock, Robert Bulander, Jennifer Rickard, James V. Harmon

**Affiliations:** a University of Minnesota Medical School, 420 Delaware St SE, Minneapolis, MN 55455, United States; b Department of Surgery, University of Minnesota, Minneapolis, MN, USA

**Keywords:** Appendicitis, Laparoscopic appendectomy, Surgical site infections, Low- and middle-income countries, Case report, AA, Acute appendicitis, LA, Laproscopic appendectomy, SSI, Surgical site infection, OA, Open appendectomy

## Abstract

**Introduction:**

Non-operative antibiotic therapy is now considered as an alternative to surgery for acute appendicitis (AA). This is in part due to the reported surgical complication rates. We report a patient who developed wound infection and port site hernia following a laparoscopic appendectomy, analyze our post-operative wound infection rates, and discuss the treatment options for AA globally.

**Presentation of case:**

We report a 40-year-old woman who developed a wound infection and subsequent port site hernia following laparoscopic appendectomy (LA) and analyze surgical site infection (SSI) and readmission rates for patients who underwent LA at our medical center. Analysis of our surveillance data demonstrated that 15/865 (1.7%) patients developed SSIs and 7/15 (47%) of these patients had positive wound cultures. Patients who developed SSIs were more likely to be male (80% vs 20%; *P* = 0.03), be older (43.0 vs 34.0; *P* = 0.04), have higher surgical wound classification scores (66.7% vs 38.2%; *P* = 0.009), and have longer operative times (82 vs 62 min; *P* = 0.003). The overall readmission rate was 2.8%.

**Discussion:**

We report a lower SSI rate after LA than usually reported. Surgical site infection following LA is rare and may be challenging to diagnose early. Additional complications such as port-site hernia may also be encountered in this setting.

**Conclusion:**

This data should inform both physicians and surgeons who must consider the expected complication rates associated with surgery for AA globally.

## Highlights

• We report a surgical site infection rate of 1.7% for laparoscopic appendectomy.
• Wound infection rates are lower for laparoscopic than for open appendectomy.
• Laparoscopic port-site infection may be followed by port-site hernias.
• Globally the laparoscopic approach is favored for appendectomy.

## 1 Introduction

Non-operative antibiotic-only therapy is now considered as efficacious as surgical management of AA [1]. Prior reports document LA surgical site infection (SSI) rates up to 5% [2]. Though we report a patient who developed a wound infection and subsequent port-site hernia, we also present data demonstrating the low SSI rate associated with LA. We advocate for the use of the LA approach globally when available where follow-up may be limited.

## 2 Presentation of case

**Fig. 1:**
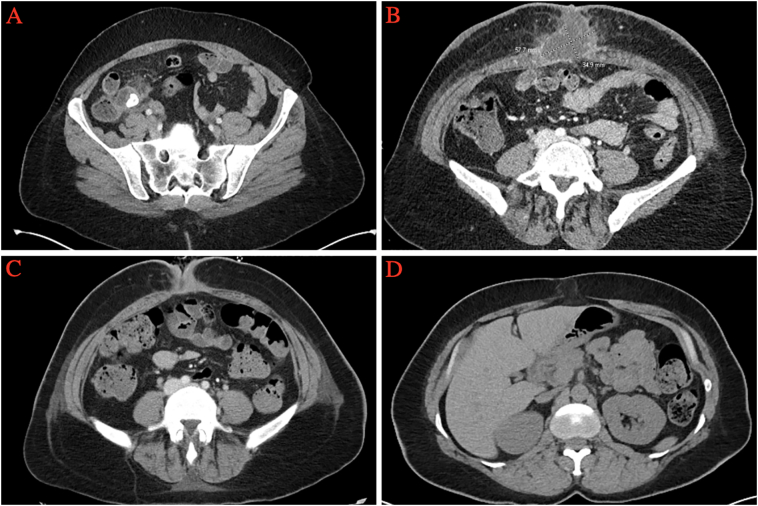
CT abdomen and pelvis with contrast. A: Enlarged appendix surrounded by inflammatory changes and a large luminal appendicolith consistent with uncomplicated AA. B: 5.8 × 3.5 cm fluid collection in the subcutaneous tissue deep to the umbilical port site incision associated with diffuse subcutaneous edema and fat stranding consistent with a laparoscopic port site wound infection. C: Post I&D imaging without evidence of an intra-abdominal fluid collection or abscess. D: A small fat-containing hernia at the laparoscopic trocar site.

**Fig. 2:**
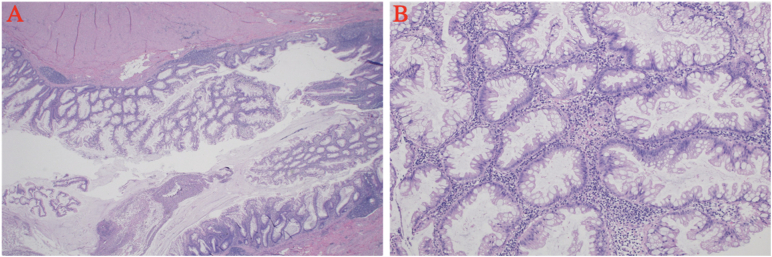
Light microscopy of the sessile serrated adenoma. A: Low power (2×) showing the appendiceal lumen with a serrated polyp arising in the mucosa. The lumen also contains fibrinopurulent exudate consistent with AA. B: High power (10×) showing the serrated and dilated mucosal glands without any cytological atypia characteristic of a sessile serrated adenoma.

We report a 40-year-old woman with a body mass index (BMI) of 38 and a history of a negative exploratory laparotomy performed in Somalia. She did not have a significant past medical history and was not taking any medications. She presented with three days of right lower quadrant abdominal pain, nausea, and anorexia. Her vital signs were normal her temperature was 98 °F, her heart rate was 90/min, her blood pressure 124/70 mm Hg, and her respiratory rate was 18/min. Physical examination revealed localized peritoneal signs in the right lower quadrant. An abdominal CT scan confirmed uncomplicated acute appendicitis (AA) and an appendicolith (Fig. 1A). The patient received 1 g ertapenem IV at the time of diagnosis and 2 g cefazolin IV at the time of anesthetic induction for laparoscopic appendectomy (LA). LA was performed 21 h after her arrival at the emergency department, and the patient was discharged home the day of surgery. Pathologic examination confirmed AA with serositis, and a sessile serrated adenoma without dysplasia was identified (Fig. 2A and B).

**Fig. 3:**
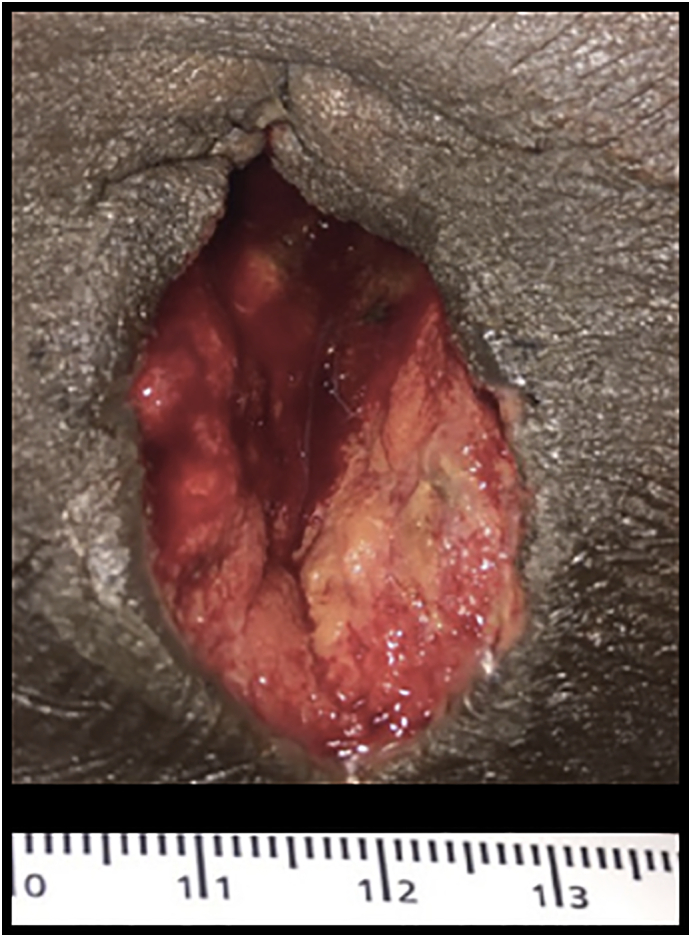
Patient's wound after incision and drainage. It was washed with 3.3% PCMX soap (Techni-Care Surgical Scrub, St. Louis, Missouri), irrigated with sterile saline, and packed open with sterile 2-inch Kerlex gauze.

On postoperative day 10 the patient presented to the surgery clinic with intense mid-abdominal pain. Vital signs were normal. Physical examination revealed tenderness, warmth, induration, and erythema at the periumbilical laparoscopic trocar incision site. The white blood cell count was 11 K (normal range 4–11). An abdominal CT scan (Fig. 1B) demonstrated a periumbilical subcutaneous fluid collection. The patient was administered IV piperacillin/tazobactam and taken to the operating room for incision and drainage. Abundant purulent fluid was drained and a wound culture was collected (Fig. 3). The midline fascia was intact, and the wound was left open and managed in the hospital due to persistent pain and surrounding cellulitis. *Klebsiella pneumoniae* and *Streptococcus constellatus* were isolated, and antibiotic coverage was narrowed to ampicillin/sulbactam. The patient's pain persisted; on POD 6, a repeat abdominal CT scan demonstrated resolution of the abscess (Fig. 1C). A 7-day course of outpatient oral ampicillin/sulbactam was provided for persistent cellulitis surrounding the open wound. The wound healed completely in ten days. Three months later, the patient presented with an incarcerated hernia at the laparoscopic trocar site. A CT scan confirmed the hernia (Fig. 1D). An open mesh hernia repair was performed. The patient's postoperative wound infection and trocar site hernia represent a Grade III surgical complication based on the Clavien-Dindo system. The patient is without further complications at 1-year postoperative follow-up.

In addition to the presentation of this case, we retrospectively analyzed surveillance data for complications, SSI, and readmission rates following LA between January 2011 and December 2017. All patients who underwent LA at our medical center during the study period were included. Excluded from analysis were patients who declined participation in research, patients converted from laparoscopic to open appendectomy (OA), and patients who underwent OA. We collected data on patient BMI, age and sex, skin preparation techniques, and operative time. We characterized wound cultures, analyzed patient outcomes, and reported superficial and organ space SSI rates in patients following LA. National Healthcare Safety Network definitions were used to evaluate 30-day postoperative infections. Categorical variables are reported as counts and percentages; continuous variables as means with standard deviations. To determine differences between cohorts, we used the χ2 test and Fisher's exact test, or Student's *t*-test, as appropriate. All *P* values are two-tailed with a significance of 0.05. We performed statistical analysis using IBM SPSS (version 25.0, Armonk, NY). Our study was approved by the University of Minnesota Institutional Review Board (IRB 1701M03261). Our work has been reported in line with the SCARE 2020 criteria [3].

**Fig. 4:**
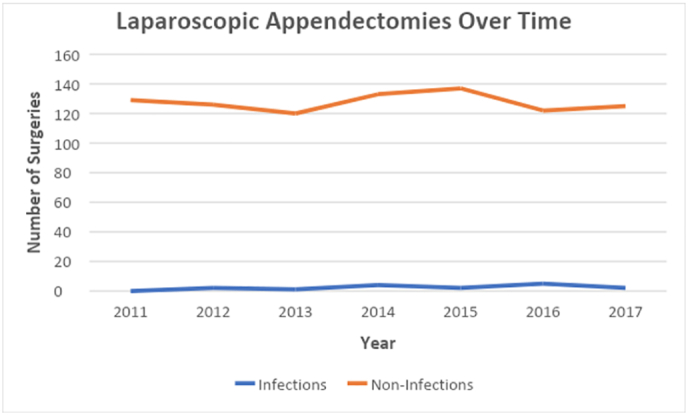
Number of SSIs after laparoscopic appendectomies performed over 7 years. Orange shows the number of patients per year without SSI. Blue shows the number of patients per year with SSI.

**Fig. 5:**
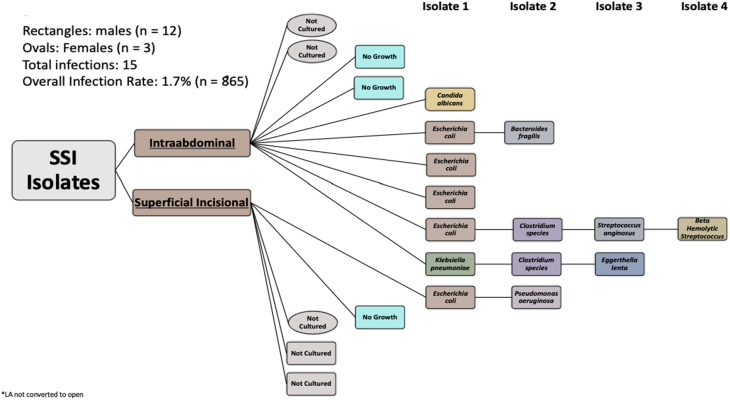
SSI pathogens isolated from wound cultures of SSIs after LA.

From 865 patients who underwent LA over a 7-year period at our medical center, we report an SSI rate of 1.7% (n = 15) (Fig. 4). Superficial SSIs were identified in 5 patients and organ space SSIs in 10 patients. The mean age of the patients in this analysis was 34 ± 15 years, BMI 26 ± 6. The American Society of Anesthesiologists (ASA) score was ≥3 in 9% of patients; 39% had a wound class ≥3. Patients who developed SSIs were more likely to be male (80% vs 20%; *P* = 0.03), to be older (43.0 vs 34.0; *P* = 0.04), have a higher surgical wound classification score (66.7% vs 38.2%; *P* = 0.009), and longer operative times (62.1 vs 81.9 min; *P* = 0.003) (Table 1). Only 47% (n = 7) of SSIs had identified isolates (Fig. 5). There were no isolates of *Staphylococcus aureus*. Thirty-nine different staff surgeons performed LAs during the study period; no individual surgeon had more than two SSIs. Chlorhexidine-gluconate operative scrub was performed in 98% of patients who did not develop an SSI and in 92% patients who di develop an SSI. The overall readmission rate was 2.8%.

**Table 1:** Variables and demographic factors associated with SSI with the *P*-values for each value are displayed.

Variables	No surgical site infections (N = 850)	Surgical site infections (SSI) (N = 15)	*P*-values
Male	446 (52.5%)	12 (80%)	0.03
Age (years)	34 ± 14.7	43 ± 15.7	0.04
BMI	26.2 ± 5.9	27.3 ± 4.9	0.473
ASA score ≥ 3	64 (7.5%)	3 (20%)	0.073
Wound class ≥ 3	325 (38.2%)	10 (66.7%)	0.009
Operative time (min)	62.1 ± 25	81.9 ± 33.5	0.003
Chlorhexidine-operative scrub	831 (97.8)	14 (92.3)	0.258

## 3 Discussion

Overestimated surgical complication rates for LA may make non-operative approaches appear erroneously advantageous for patients with AA. Although the patient we report developed a wound infection and a subsequent port-site hernia, we present data demonstrating that LA is associated with a lower SSI rate than typically reported. Our single-center retrospective review confirms that LA is a safe and effective option for the treatment of AA, with an SSI rate of 1.7%. In terms of the surgical management of AA, one of the many advantages of LA over OA is the significantly lower SSI rates in patients undergoing LA. The World Health Organization (WHO) performed a meta-analysis of the incidence of SSI after appendectomy among low-, middle-, and high-income countries around the world and found that LA was associated with an SSI rate of 4.6%, while OA was associated with an SSI rate of 11% [2]. Multiple centers have reported low SSI rates after LA compared to OA due to smaller incisions and the use of an extraction bag which reduces the exposure of the infected appendix to the skin and subcutaneous tissue [4].

Prior publications describing the treatment of AA using antibiotics alone have reported the need for appendectomy after failed medical management with subsequent high rates of surgical complications, possibly due to delayed appropriate surgical interventions. A 5-year follow-up report of a European trial of patients treated with antibiotics alone for AA reported that 38.9% of these patients required LA [5]. Similarly, a United States randomized control trial reported that 29% of patients treated with antibiotics alone required an appendectomy within 90 days of follow-up [1]. The 2021 CODA trial results support non-operative antibiotic treatment of AA as not inferior to surgery [6].

When SSIs do occur, the Surgical Infection Society guidelines on the management of intra-abdominal infection suggest using antimicrobial agents against gram-negative *Enterobacteriaceae*, *aerobic streptococci*, and obligate anaerobic microorganisms [7]. As such, the patient described in our case report received 1 g ertapenem in the ED and 2 g cefazolin peri-operatively. For patients with uncomplicated appendicitis, most surgeons recommend against the administration of additional postoperative antibiotics [8]. The STOP-IT trial demonstrated that a 4-day antibiotic course resulted in equivalent patient outcomes as an 8-day course [9]; and other authors confirm that a short antibiotic course was as effective as a long course [10].

There are no uniform guidelines for the management of AA on a global scale. In austere environments, where facilities and personnel are limited, non-operative treatment may be the most practical option. When access to antibiotics is limited and patients must travel great distances for follow-up, non-operative management may not be the safest option. An extensive meta-analysis demonstrates that LA is associated with lower SSI rates than OA in low- and middle- income countries [11]. Without systems in place to evaluate surveillance, infection prevention, and antimicrobial treatment, it is increasingly difficult to manage surgical infections [12]. A recent meta-analysis assessed that, while the overall global incidence of SSI after appendectomy was 7.0%, the incidence of SSI was higher in low-income countries (11.1%) compared to high-income countries (6.2%) [2]. Factors associated with lower rates of SSI in low- and middle-income countries include early surgical intervention and living in close proximity to a hospital [11], [13]. Following the LA, the reported low rates of SSI may diminish the global disease burden due to AA.

Limitations of our retrospective review include that this is a single-center analysis of SSI surveillance data. Although we maintain a robust SSI surveillance program, patients may have presented to outside facilities with SSIs consequently, their data was not included in our analysis.

## 4 Conclusion

We report a patient who developed additional complications that followed the occurrence of a surgical site infection but did identify a serrated sessile appendiceal polyp. Our review confirms a low rate of SSIs following LA, which remains a safe and effective option for the definitive treatment of uncomplicated AA.

## Provenance and peer review

Not commissioned, externally peer-reviewed.

## Consent

Written informed consent was obtained from the patient for publication of this case report and accompanying images. A copy of the written consent is available for review by the Editor-in-Chief of this journal on request.

## Ethical approval

Our study was approved by our Institutional Review Board (IRB number 1701M03261).

## Funding

This research did not receive any specific grant from funding agencies in the public, commercial, or not-for-profit sectors.

## Guarantor

James V Harmon.

## Research registration number

N/A.

## CRediT authorship contribution statement

- Katie Jeddeloh: Responsible for the data analysis/interpretation and writing of the manuscript.
- Jena Velji-Ibrahim: Responsible for the data analysis/interpretation and writing of the manuscript.
- Emily Stock: Responsible for study design, data collection, and data analysis/interpretation.
- Robert Bulander: Responsible for study design and data analysis/interpretation.
- Jennifer Rickard: Responsible for writing the manuscript.
- James V Harmon: Responsible for study design, data analysis/interpretation, and writing of the manuscript.

## Declaration of competing interest

The authors have no other relationships, conditions, or circumstances that present a potential conflict of interest. The authors alone are responsible for the content and writing of this article.

## References

[1] Flum D.R. (2015). Acute appendicitis — appendectomy or the “antibiotics first” strategy. N. Engl. J. Med..

[2] Danwang C., Bigna J.J., Tochie J.N., Mbonda A., Mbanga C.M., Nzalie R.N.T. (2020). Global incidence of surgical site infection after appendectomy: a systematic review and meta-analysis. BMJ Open.

[3] Agha R.A., Franchi T., Sohrabi C., Mathew G., for the SCARE Group (2020). The SCARE 2020 guideline: updating consensus Surgical CAse REport (SCARE) guidelines. Int. J. Surg..

[4] Suh Y.J., Jeong S.Y., Park K.J., Park J.G., Kang S.B., Kim D.W. (2012). Comparison of surgical-site infection between open and laparoscopic appendectomy. J. Korean Surg. Soc..

[5] Salminen P., Tuominen R., Paajanen H., Rautio T., Nordström P., Aarnio M. (2018). Five-year follow-up of antibiotic therapy for uncomplicated AA in the APPAC randomized clinical trial. JAMA J. Am. Med. Assoc..

[6] Flum D.R., Davidson G.H., Monsell S.E., Shapiro N.I., Odom S.R., Sanchez S.E. (2020). A randomized trial comparing antibiotics with appendectomy for appendicitis. N. Engl. J. Med..

[7] Mazuski J.E., Tessier J.M., May A.K., Sawyer R.G., Nadler E.P., Rosengart M.R. (2017). The surgical infection society revised guidelines on the management of intra-abdominal infection. Surg. Infect..

[8] Berriós-Torres S.I., Umscheid C.A., Bratzler D.W., Leas B., Stone E.C., Kelz R.R. (2017). Centers for disease control and prevention guideline for the prevention of surgical site infection, 2017. JAMA Surg..

[9] Sawyer R.G., Claridge J.A., Nathens A.B., Rotstein O.D., Duane T.M., Evans H.L. (2015). Trial of short-course antimicrobial therapy for intraabdominal infection. N. Engl. J. Med..

[10] Kroon H.M., Kenyon-Smith T., Nair G., Virgin J., Thomas B., Juszczyk K. (2021). Safety and efficacy of short-course intravenous antibiotics after complicated appendicitis in selected patients. Acta Chir. Belg..

[11] Foster D., Kethman W., Cai L.Z., Weiser T.G., Forrester J.D. (2018). Surgical site infections after appendectomy performed in low and middle human development-index countries: a systematic review. Surg. Infect..

[12] Rickard J., Beilman G., Forrester J., Sawyer R., Stephen A., Weiser T.G. (2019). Surgical infections in low- and middle-income countries: a global assessment of the burden and management needs. Surg. Infect..

[13] Melese Ayele W. (2021). Prevalence of postoperative unfavorable outcome and associated factors in patients with appendicitis: a cross-sectional study. Open Access Emerg. Med..

